# Biofilm-Mediated Antimicrobial Resistance in Pediatric *Klebsiella pneumoniae* Urinary Tract Infections: A Narrative Review of Mechanisms, Clinical Challenges, and Therapeutic Strategies

**DOI:** 10.3390/antibiotics15080783

**Published:** 2026-08-13

**Authors:** Larisa Goroftei, Cristina-Mihaela Popescu, Irina Profir, Geanina-Adelina Jalba, Gabriela Gurau

**Affiliations:** 1 Medical and Pharmaceutical Research Center, Faculty of Medicine and Pharmacy, “Dunărea de Jos” University of Galați, 800008 Galati, Romania; larisa.goroftei@ugal.ro (L.G.); cristina.popescu@ugal.ro (C.-M.P.); geanina.sirbu@ugal.ro (G.-A.J.); gabriela.gurau@ugal.ro (G.G.); 2 Clinical Medical Department, Faculty of Medicine and Pharmacy, “Dunărea de Jos” University of Galați, 800216 Galati, Romania; 3 “Sf. Ioan” Clinical Emergency Pediatric Hospital, 800487 Galati, Romania; 4 Department of Dental Medicine, Faculty of Medicine and Pharmacy, “Dunărea de Jos” University of Galați, 800201 Galati, Romania; 5 Department of Morphology and Functional Sciences, Faculty of Medicine and Pharmacy, “Dunărea de Jos” University of Galați, 800008 Galati, Romania

**Keywords:** anti-bacterial agents, bacteriophages, biofilms, child, drug resistance, *Klebsiella pneumoniae*, quorum sensing, urinary tract infections

## Abstract

Urinary tract infections (UTIs) caused by *Klebsiella pneumoniae* are an increasing challenge in pediatric practice due to the combined effects of biofilm formation, multidrug resistance (MDR), and limited therapeutic options for children. Biofilm development promotes bacterial persistence by impairing antibiotic penetration, enabling metabolic adaptation, promoting persister-cell formation, facilitating horizontal gene transfer (HGT), and inducing stress-induced mutagenesis, thereby reducing the effectiveness of conventional antimicrobial therapy. These mechanisms are further compounded by pediatric-specific challenges, including age-dependent pharmacokinetic variability, congenital urinary tract abnormalities, device-associated infections, and the limited availability of validated diagnostic tools for biofilm-associated infections. This narrative review integrates current knowledge of the molecular mechanisms underlying biofilm-mediated antimicrobial resistance with the unique diagnostic, pharmacological, and therapeutic challenges encountered in pediatric patients with *K. pneumoniae* UTIs. Emerging therapeutic strategies, such as optimized antibiotic combination therapy, bacteriophages, biofilm matrix-degrading enzymes, quorum-sensing inhibitors (QSIs), antimicrobial peptides (AMPs), and microbiome-directed approaches are critically evaluated with particular emphasis on their potential applicability in children. Although several anti-biofilm strategies have demonstrated encouraging results in experimental models, robust pediatric clinical evidence remains scarce. Current international guidelines continue to rely primarily on planktonic antimicrobial susceptibility testing without addressing biofilm-specific therapeutic considerations. In the absence of validated biofilm diagnostics, catheter stewardship and dosing optimization remain the most defensible clinical interventions available today. Broader translation of anti-biofilm strategies into pediatric practice will require dedicated pharmacokinetic studies, standardized biofilm diagnostics, and prospective clinical trials.

## 1. Introduction

Urinary tract infections (UTIs) are among the most common bacterial infections in childhood, accounting for a substantial proportion of febrile illnesses in infants and young children and representing one of the leading indications for antibiotic prescribing in pediatric practice [1,2,3,4]. When inadequately treated or recurrent, UTIs may result in long-term renal complications, including renal scarring, hypertension, and chronic kidney disease [1,5,6]. Although *Escherichia coli* (*E. coli*) remains the predominant uropathogen, *Klebsiella pneumoniae* (*K. pneumoniae*) consistently ranks second among the most frequently isolated pathogens in pediatric UTIs [7,8,9,10]. Its increasing clinical importance is largely attributable to its remarkable ability to acquire and disseminate antimicrobial resistance determinants, including extended-spectrum β-lactamases (ESBLs) and carbapenemases, thereby limiting available therapeutic options [11,12,13,14].

Beyond its prevalence, *K. pneumoniae* possesses a unique capacity to establish persistent UTIs through biofilm formation [15,16,17]. Biofilms are highly organized bacterial communities enclosed within a self-produced extracellular polymeric substance (EPS) matrix that adheres to both biotic surfaces, such as the urothelium, and abiotic surfaces, such as urinary catheters [18]. In pediatric patients, the high prevalence of congenital anomalies of the kidney and urinary tract (CAKUT), together with the frequent need for urinary catheterization in hospitalized children, provides favorable conditions for biofilm development and persistence [5,19].

Biofilm formation profoundly alters bacterial susceptibility to antimicrobial therapy. Rather than acting through a single mechanism, biofilms confer protection through multiple complementary processes. They include reduced antibiotic penetration by the EPS matrix, the emergence of metabolically dormant persister cells, and enhanced horizontal gene transfer (HGT), which facilitates the dissemination of resistance determinants [18,20,21,22,23]. Collectively, these mechanisms transform otherwise treatable infections into persistent, recurrent, and therapeutically challenging conditions.

Although the molecular mechanisms underlying biofilm-mediated antimicrobial resistance have been extensively investigated, most available evidence originates from adult populations or experimental models. Consequently, important questions remain regarding their clinical relevance in pediatric UTIs, where physiological differences, age-dependent pharmacokinetics and pharmacodynamics, limited availability of approved antimicrobial agents, and increased susceptibility to drug toxicity substantially influence therapeutic decision-making [19,24,25,26]. Furthermore, current pediatric UTI guidelines do not specifically address the diagnosis or management of biofilm-associated infections, highlighting an important gap between experimental evidence and clinical practice [19,24]. Furthermore, clinicians currently have no guideline-endorsed basis for distinguishing a biofilm-associated infection from a straightforward planktonic one at the bedside. Treatment decisions default to susceptibility data that may not reflect the organism’s behavior in situ, creating a mismatch that plausibly contributes to therapeutic failures and recurrences detailed in Section 4 despite apparent in vitro activity [19,24]. In the absence of validated bedside diagnostics, two measures are already available to clinicians: prompt removal of unnecessary urinary catheters and antimicrobial stewardship targeting sub-therapeutic dosing. They represent the most immediately actionable responses to this uncertainty and are both discussed in Section 5.1.

This narrative review integrates current evidence on the molecular mechanisms underlying biofilm-mediated antibiotic resistance in pediatric UTIs caused by *K. pneumoniae*, discusses the unique clinical challenges encountered in children, critically evaluates emerging antibiofilm therapeutic strategies, and highlights the major translational gaps that currently limit their incorporation into pediatric clinical practice. The mechanistic and adult-focused biofilm literature synthesized here is largely established; this review’s distinct contribution is its integration into a pediatric clinical and translational framework, an area that remains comparatively underdeveloped despite existing broad reviews of *K. pneumoniae* UTI and of adult-focused antibiofilm strategies.

**Figure 1:**
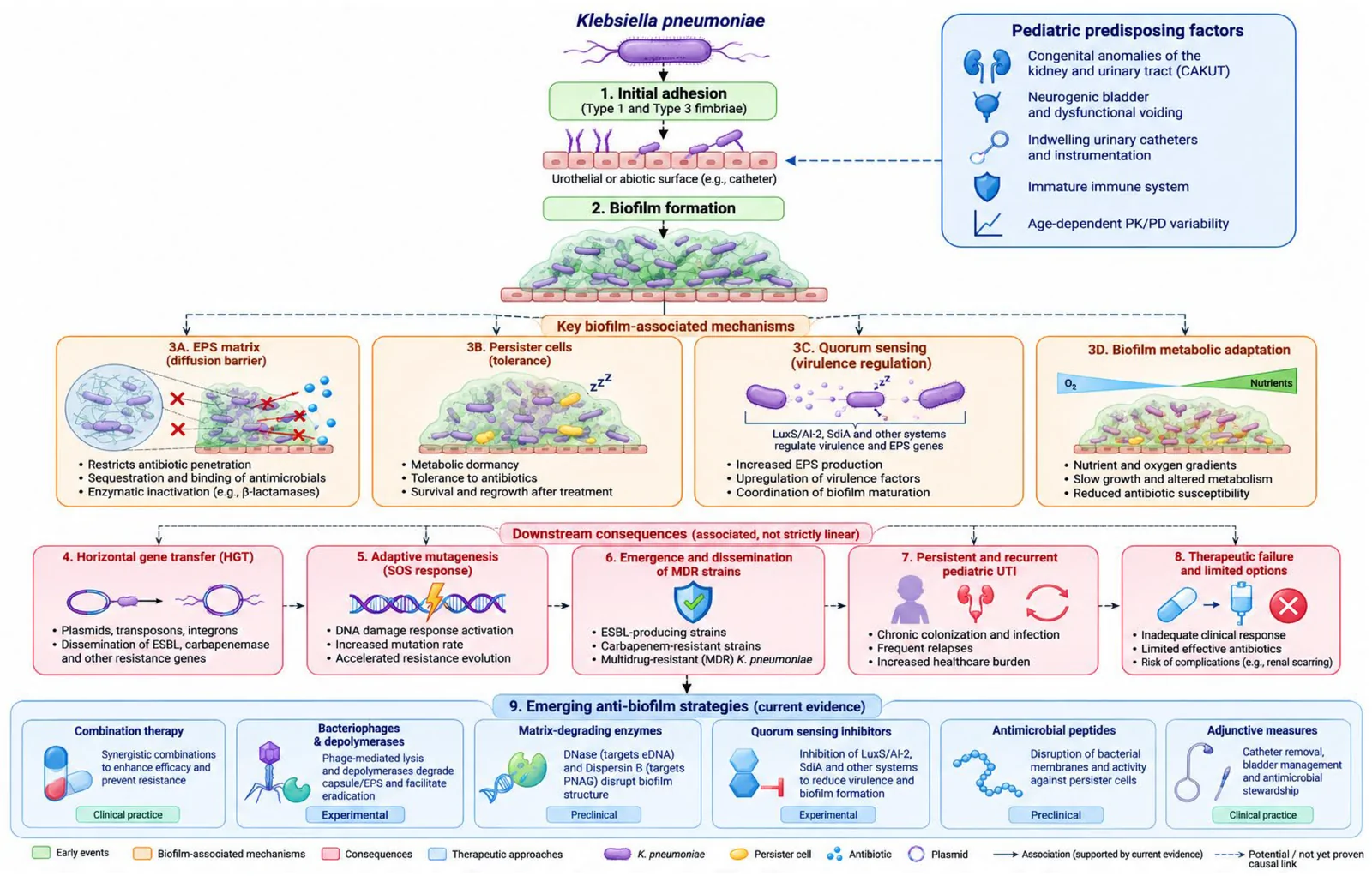
Overview of the molecular mechanisms underlying biofilm-mediated antimicrobial resistance in pediatric *K. pneumoniae* UTIs. Initial adhesion via type 1 and type 3 fimbriae promotes biofilm formation, leading to EPS production, persister cell formation, quorum-sensing (QS) activation, and metabolic adaptation. These processes facilitate HGT, adaptive mutagenesis, and multidrug resistance, ultimately contributing to recurrent infections and therapeutic failure. Emerging anti-biofilm strategies target different stages of this pathogenic cascade. Arrows indicate associations based on available experimental and clinical data and do not imply a strict linear or proven causal relationship.

The interplay among mechanisms underlying biofilm-mediated antimicrobial resistance is summarized schematically in Figure 1.

## 2. Search Strategy

A literature search was conducted in PubMed/MEDLINE, with the primary window restricted to articles published between 2021 and 2026 for clinical, epidemiological, and therapeutic literature; foundational mechanistic studies published before 2021 were additionally included where necessary to provide biological context for well-established biofilm processes (e.g., fimbrial adhesion, QS regulation, and EPS matrix composition). The search combined terms related to *K. pneumoniae*, urinary tract infection, and pediatric populations (child, pediatric, paediatric, infant, neonate), further stratified by resistance phenotype (multidrug-resistant, extended-spectrum β-lactamase-producing, carbapenem-resistant), biofilm-associated mechanisms (quorum sensing, persister cells, poly-β-1,6-N-acetylglucosamine synthesis, HGT), and therapeutic considerations (pharmacokinetic/pharmacodynamic dosing, combination therapy, antimicrobial stewardship). Boolean operators were used to construct each query, with wildcard truncation applied to capture variant terminology.

Study selection was investigator-driven and guided by relevance to the review’s three thematic pillars: molecular biofilm mechanisms, pediatric clinical challenges, and emerging therapeutic strategies. We focused on the three directions rather than abide by a predefined systematic protocol, consistent with standard narrative review methodology. Given the scarcity of pediatric-specific data on biofilm-associated *K. pneumoniae* UTI, foundational mechanistic and in vitro evidence from the broader *K. pneumoniae* biofilm literature was incorporated where necessary to construct a coherent framework, and is identified as such throughout the text.

## 3. Molecular Mechanisms

### 3.1. Biofilm Formation and Maturation in Klebsiella pneumoniae

Biofilm formation in *K. pneumoniae* is a dynamic, highly regulated process that begins with bacterial adhesion to biotic or abiotic surfaces. Two functionally distinct fimbrial systems primarily mediate this initial attachment. Type 1 fimbriae promote adhesion to uroepithelial cells and urinary matrix proteins, particularly Tamm–Horsfall glycoprotein, thereby facilitating early colonization of the urinary tract [27]. In contrast, type 3 fimbriae primarily mediate interbacterial aggregation and attachment to abiotic surfaces, including urinary catheters, and play a pivotal role in biofilm maturation and structural stability [27,28,29,30].

Following adhesion, bacterial proliferation is accompanied by EPS production, which encapsulates the developing bacterial community and promotes the formation of microcolonies [31,32]. Continued EPS synthesis, together with bacterial replication, results in the maturation of a complex three-dimensional biofilm characterized by interconnected water channels that facilitate nutrient distribution and waste removal, thereby maintaining bacterial viability throughout the biofilm architecture [18].

The distinct functions of type 1 and type 3 fimbriae highlight *K. pneumoniae*’s adaptive capacity to colonize both host tissues and medical devices, underscoring biofilm formation as a central virulence strategy in pediatric UTIs. Importantly, the mature biofilm establishes the structural and metabolic heterogeneity that underlies the antimicrobial tolerance mechanisms discussed in the next section.

### 3.2. Diffusion Barrier and Biofilm Microenvironment

The EPS matrix represents the principal structural component of the biofilm and constitutes one of the earliest barriers limiting antimicrobial activity [33,34]. Rather than functioning solely as a physical scaffold, the EPS matrix restricts antibiotic diffusion, modifies local drug concentrations, and creates a protective microenvironment that promotes bacterial survival [18]. In addition, negatively charged matrix components may sequester cationic antibiotics, particularly aminoglycosides, while locally concentrated β-lactamases further reduce the activity of β-lactam antibiotics [18,35,36].

Importantly, antibiotic penetration varies substantially depending on the physicochemical properties of individual agents. As synthesized by Uruén et al., experimental studies demonstrated that aminoglycoside diffusion is markedly impaired in mature *K. pneumoniae* biofilms, whereas fosfomycin retains greater penetration due to its low molecular weight and hydrophilic properties [18]. These findings emphasize that antimicrobial susceptibility determined under planktonic conditions does not accurately predict drug activity within mature biofilms. Consequently, conventional minimum inhibitory concentration (MIC) testing may underestimate the antibiotic concentrations required for effective treatment of biofilm-associated infections, a limitation highlighted by Zdarska et al. and Wang et al. [37,38].

Beyond restricting antibiotic penetration, biofilms generate pronounced oxygen and nutrient gradients, creating metabolically heterogeneous bacterial populations. Cells located in deeper biofilm layers adopt a slow-growing or dormant phenotype, giving rise to persister cells that exhibit transient tolerance to antibiotics targeting active cellular processes [18,39]. Because β-lactams require active cell-wall synthesis, fluoro-quinolones depend on ongoing deoxyribonucleic acid (DNA) replication, and aminoglycosides require active protein synthesis, their bactericidal activity is substantially reduced against these metabolically inactive cells [18,39]. As a result, persister cells frequently survive antimicrobial therapy and may repopulate the biofilm after treatment cessation, providing a mechanistic explanation for recurrent infections observed in clinical practice [40,41].

### 3.3. Adaptive Response of Biofilm to Antibiotic Stress

Exposure to sub-inhibitory concentrations of antibiotics not only exerts selective pressure on *K. pneumoniae* but also induces adaptive responses that promote biofilm persistence and antimicrobial tolerance. Experimental studies have shown that sub-inhibitory antibiotic exposure upregulates genes involved in EPS biosynthesis, leading to a denser, less permeable biofilm matrix [18,27]. Rather than weakening the bacterial community, antibiotic exposure may therefore paradoxically enhance biofilm resilience.

Importantly, adaptive responses appear to differ across antibiotic classes. β-lactams predominantly stimulate the expression of genes involved in EPS production, thereby reinforcing the diffusion barrier, whereas aminoglycosides more readily promote persister-cell formation through tolerance-associated pathways [18,42]. Although both mechanisms ultimately reduce antimicrobial efficacy, they reflect distinct bacterial survival strategies, highlighting the complexity of biofilm adaptation under antibiotic stress.

In addition to these phenotypic adaptations, antibiotic-induced DNA damage activates the SOS response in biofilm-associated bacteria, promoting the expression of error-prone DNA polymerases and increasing mutation rates [18]. France et al. demonstrated that this adaptive response facilitates the emergence and selection of heritable resistance mutations within densely populated biofilm communities [42]. However, most evidence supporting these mechanisms derives from experimental biofilm models. Their contribution to treatment failure in vivo, particularly in pediatric UTIs, remains poorly defined because clinical studies directly evaluating biofilm-associated transcriptional responses are currently lacking. This knowledge gap underscores the need for translational studies that link experimental observations to clinically relevant outcomes.

### 3.4. Biofilm as an Amplifier of Genetic Resistance and the Role of Quorum Sensing

Beyond protecting bacteria from antimicrobial exposure, biofilms create an environment that facilitates HGT, thereby accelerating the dissemination of antimicrobial resistance determinants [43,44]. The high bacterial density, prolonged cell-to-cell contact, and protective EPS matrix promote plasmid exchange through bacterial conjugation. This process is particularly relevant in *K. pneumoniae*, which efficiently acquires and disseminates plasmids encoding ESBLs, including CTX-M enzymes, as well as carbapenemases such as *Klebsiella pneumoniae* Carbapenemase (KPC), New Delhi metallo-β-lactamase (NDM), and Oxacillinase-48 (OXA-48)-like variants [35,45,46,47]. Consequently, biofilms function not only as protective structures but also as reservoirs that facilitate the emergence and spread of multidrug-resistant (MDR) and extensively drug-resistant (XDR) phenotypes [48].

Biofilm development is further coordinated by quorum sensing (QS), a cell-density-dependent communication system that regulates bacterial collective behavior [49,50]. In *K. pneumoniae*, two major regulatory pathways have been described. The LuxS/Autoinducer-2 (AI-2) system primarily controls biofilm architecture by regulating transcription of genes involved in extracellular polysaccharide (EPS) synthesis, including those encoding EPS and poly-β-1,6-N-acetyl-D-glucosamine (PNAG) [49,50]. In contrast, the orphan LuxR-family receptor SdiA responds to exogenous acyl-homoserine lactones produced by neighboring bacterial species and modulates the expression of type 1 fimbriae and other genes involved in biofilm maturation [28].

Although these regulatory pathways have distinct biological functions, their relative contribution to biofilm persistence during UTI remains incompletely understood. Current evidence derives predominantly from experimental studies, and whether inhibition of individual QS pathways is sufficient to disrupt mature biofilms in vivo, particularly in pediatric infections, has not yet been established [28,51]. Nevertheless, the coordinated regulation of extracellular matrix production, adhesion, and virulence factor expression highlights QS as a promising anti-biofilm therapeutic target [49,50,52]. Emerging QS-targeted therapeutic strategies are discussed in Section 5.

Table 1 summarizes the principal mechanisms of biofilm-mediated antibiotic resistance discussed in this section, together with their clinical implications and corresponding therapeutic targets.

### 3.5. Sensing Hypervirulent K. pneumoniae: Biofilm and the Convergence of Virulence and Resistance

An additional dimension relevant to the biofilm framework described above is the emergence of hypervirulent *K. pneumoniae* (hvKP), a subtype distinguished from classical *K. pneumoniae* (cKP) by a hypermucoviscous phenotype and characteristic virulence determinants, including the regulatory genes rmpA and rmpA2 and the aerobactin siderophore system (iuc). Although hvKP has classically been defined by non-urinary manifestations, its relevance to this review lies in the convergence of virulence and resistance mechanisms described below, which is increasingly reported to extend to the urinary tract in both adults and, more recently, children. Historically, it was associated with severe community-acquired infections such as pyogenic liver abscess, endophthalmitis, and metastatic infection in otherwise immunocompetent hosts [15]. Although less common than in adults, pediatric hvKP infections are increasingly reported, with case series describing hvKP-associated bacteremic pyogenic liver abscess and pneumonia in children and adolescents, including fatal outcomes despite apparent in vitro antimicrobial susceptibility [53,54].

Critically, hvKP does not represent a uniformly attenuated biofilm phenotype: biofilm-forming ability among *K. pneumoniae* isolates has been correlated with the same regulatory and structural genes implicated in classical biofilm biology (luxS, mrkA, pgaA), with hypermucoviscosity-associated determinants (rmpA, rmpA2, iuc) co-occurring in a subset of biofilm-forming strains [55].

Of particular concern is the growing convergence of the hvKP and MDR phenotypes. It was once considered largely mutually exclusive owing to a fitness trade-off between capsule overproduction and plasmid maintenance. Now, there is increasing documentation of the acquisition of MDR plasmids by hypervirulent lineages or of virulence plasmids by classical MDR lineages [15,56]. Convergent MDR-hvKP strains combine the therapeutic limitations of classical CRKP biofilm infection with the systemic and metastatic potential of hvKP. To date, this convergence has been documented in the urinary tract in a single reported case, in which adaptive evolution toward this phenotype was observed during one patient’s treatment [17]. Broader pediatric urinary evidence for this convergence is currently lacking. Routine microbiological workup does not currently differentiate hvKP from cKP at the bedside, and clinicians managing *K. pneumoniae* UTI in children should maintain a heightened index of suspicion for hvKP where bacteremia, unexplained abscess formation, or infection without typical anatomical predisposing factors is present.

## 4. Specific Clinical Challenges in the Management of Pediatric UTIs with *Klebsiella pneumoniae* Biofilm

Translating laboratory knowledge of biofilm-mediated resistance into effective pediatric clinical practice reveals a series of complex, often underestimated challenges. The management of these infections in children extends well beyond selecting an in vitro-active antibiotic, requiring a holistic approach that accounts for host-specific physiological particularities, the inherent limitations of standard diagnostic tools, and a therapeutic landscape increasingly constrained by the emergence of MDR and XDR phenotypes.

### 4.1. Diagnostic and Monitoring Difficulties

Conventional urine culture remains the cornerstone of UTI diagnosis; however, its diagnostic performance is substantially reduced in biofilm-associated infections [57,58]. Because routine culture detects only planktonic bacteria present in urine, it may underestimate the true bacterial burden or even yield false-negative results when microorganisms are predominantly embedded within biofilms attached to the urothelium or urinary catheters [59,60]. This limitation is particularly relevant for *K. pneumoniae*, as biofilm-forming isolates are frequently associated with MDR phenotypes and commonly harbor virulence and resistance determinants such as pgaA, mrkA, luxS, and carbapenemase-encoding bla genes [55]. As noted in Section 3.2, this discordance is directly relevant here [37,38].

Diagnostic uncertainty is even greater in neonates and young infants. Although conventional urine culture successfully identifies the causative pathogen in most febrile infants, *K. pneumoniae* remains an important, albeit less frequent, uropathogen in this age group [9,61]. Molecular diagnostic approaches, particularly 16S ribosomal RNA sequencing, have demonstrated greater sensitivity for pathogen detection than conventional culture methods [62]. However, their limited specificity currently restricts routine clinical implementation [62]. Consequently, distinguishing persistent infection from relapse or accurately identifying the causative organism remains challenging, particularly during early infancy [62,63].

Several techniques, including confocal laser scanning microscopy (CLSM), Congo red agar assays, and microtiter plate biofilm assays, enable direct assessment of biofilm formation under experimental conditions [55,60]. Nevertheless, these methods remain confined to research laboratories and have not been standardized for routine clinical practice. As a result, clinicians currently lack validated diagnostic tools to confirm biofilm-associated infection in individual pediatric patients.

Collectively, these diagnostic limitations contribute to delayed recognition of persistent infection, uncertainty regarding treatment success, and difficulty distinguishing microbiological cure from persister-cell-mediated relapse. Addressing these challenges will likely require integrating molecular diagnostics with the development of clinically applicable biomarkers to identify biofilm-associated infections. Table 2 summarizes the diagnostic approaches discussed above, together with their main limitations, clinical implications, and current status in pediatric practice.

### 4.2. Failure of Conventional Antibiotic Therapy

The most important clinical consequence of biofilm formation in *K. pneumoniae* UTIs is therapeutic failure despite apparent in vitro susceptibility [64,65]. As discussed in Section 3.2, EPS-mediated penetration failure and metabolic persistence jointly explain this pattern. Together with the strong association between biofilm formation and MDR, these mechanisms substantially reduce the effectiveness of conventional antimicrobial therapy [18,65].

Recent surveillance studies consistently indicate an increasing prevalence of MDR *K. pneumoniae* among pediatric urinary isolates across different geographic regions [11,66,67,68,69,70,71,72,73,74,75]. Although reported MDR rates vary between studies, the overall trend is remarkably consistent. Hospitalized children, particularly those with recent antibiotic exposure or recurrent UTIs, are at the highest risk of infection caused by MDR organisms [76,77]. Longitudinal surveillance further demonstrates a progressive increase in resistance over the last decade, emphasizing that antimicrobial resistance is an evolving rather than static phenomenon [76].

The emergence of ESBL-producing *K. pneumoniae* has further narrowed therapeutic options. In a five-year Taiwanese cohort, ESBL-producing *Enterobacteriaceae* accounted for 14.1% of pediatric UTIs, with resistance increasing throughout the study period [13]. ESBL-positive isolates frequently exhibited co-resistance to gentamicin, trimethoprim–sulfamethoxazole, and ciprofloxacin, leaving carbapenems and amikacin as the most consistently active agents [13]. The practical therapeutic implication of ESBL co-resistance is profound: it abolishes the utility of the most commonly prescribed empirical oral and intravenous regimens, compressing therapeutic options to agents with significant toxicity concerns in children [78,79,80,81,82]. Although fosfomycin and nitrofurantoin have been proposed as oral alternatives for uncomplicated lower UTIs, their activity against *K. pneumoniae* remains substantially lower than that observed for *E. coli*, limiting the direct extrapolation of treatment strategies between these two pathogens [83]. Collectively, these findings illustrate that antimicrobial susceptibility patterns established for *E. coli* should not automatically guide the management of pediatric *K. pneumoniae* infections.

Fluoroquinolones provide another example of the discrepancy between theoretical antimicrobial activity and clinical applicability. Although these agents demonstrate relatively favorable biofilm penetration, their routine use in children remains restricted due to concerns about musculoskeletal toxicity [84]. Furthermore, transferable quinolone resistance mechanisms, including aac(6’)-Ib-cr, qnrB, and qnrS, are increasingly detected among pediatric *K. pneumoniae* isolates and frequently coexist with ESBL genes, further limiting the usefulness of this antibiotic class [85].

At the highest end of the resistance spectrum, carbapenem-resistant *K. pneumoniae* (CRKP) has emerged as a major concern in neonatal intensive care units (NICUs) [56,86,87,88]. Genomic surveillance has demonstrated the dissemination of highly resistant clones carrying transferable carbapenemase-encoding plasmids, illustrating CRKP’s capacity to persist for prolonged periods within healthcare settings [14]. Encouragingly, long-term antimicrobial stewardship interventions have shown that reducing broad-spectrum antibiotic exposure can significantly decrease MDR *K. pneumoniae* isolation rates among preterm infants, confirming that antibiotic pressure remains a modifiable driver of resistance [89].

Overall, current evidence indicates that therapeutic failure in pediatric *K. pneumoniae* UTIs cannot be explained solely by antimicrobial resistance or biofilm formation in isolation. Rather, it results from the interaction between biofilm-mediated tolerance, the progressive accumulation of acquired resistance mechanisms, and the limited number of antimicrobial agents suitable for pediatric patients. This convergence substantially restricts effective treatment options and highlights the need for therapeutic strategies specifically targeting biofilm-associated infections. Table 3 summarizes the principal clinical challenges discussed in this section, along with supporting evidence and their implications for management.

### 4.3. Unique Challenges Related to the Pediatric Host

Beyond the microbiological complexity of *K. pneumoniae* biofilms, the physiological and anatomical characteristics of pediatric patients introduce additional challenges that substantially influence antimicrobial therapy and clinical outcomes. Age-dependent maturation of organ systems, congenital urinary tract abnormalities, and the limited availability of antimicrobial agents approved for pediatric use collectively distinguish the management of biofilm-associated UTIs in children from that in adults.

#### 4.3.1. Pharmacokinetic and Pharmacodynamic Considerations

Children cannot be considered pharmacological equivalents of adults because drug disposition changes continuously throughout growth and development [90,91,92]. Age-related differences in gastrointestinal absorption, body composition, protein binding, hepatic metabolism, and renal elimination produce highly variable pharmacokinetic profiles, particularly in neonates and young infants, whose physiological functions remain incompletely mature [93,94,95].

These developmental differences complicate antimicrobial therapy in biofilm-associated infections. Effective treatment requires not only achieving concentrations exceeding the MIC for planktonic bacteria but also maintaining drug exposure sufficient to penetrate the biofilm matrix and reach metabolically inactive bacterial subpopulations. Experimental evidence suggests that these concentrations may substantially exceed those required to inhibit planktonic organisms [64,96]. Consequently, standard adult dosing regimens cannot be directly extrapolated to pediatric patients and frequently require individualized, age- and weight-adjusted dosing strategies.

This therapeutic challenge is particularly evident with antibiotics that have narrow therapeutic windows. Aminoglycosides remain important agents against MDR Gram-negative pathogens; however, their use is constrained by well-recognized nephrotoxicity and ototoxicity, especially in neonates and critically ill children [97,98]. Similarly, colistin remains an important rescue therapy for XDR infections despite the lack of well-defined pediatric dosing recommendations. In a single-center, retrospective cohort of 178 Thai pediatric patients, the nephrotoxicity rate was 29.8% [99]. However, this percentage should not be generalized to a fixed population rate given the paucity of comparable pediatric studies [100,101]. These observations illustrate the inherent difficulty of balancing adequate antimicrobial exposure against acceptable toxicity in children with biofilm-associated infections.

The limited availability of pediatric pharmacokinetic/pharmacodynamic (PK/PD) data represents an additional obstacle to optimal therapy, particularly for newer β-lactam/β-lactamase inhibitor (BLI) combinations active against CRKP [102,103]. Because developmental physiology substantially influences drug disposition, adult PK models cannot be reliably extrapolated to children, highlighting the need for dedicated pediatric population pharmacokinetic studies and pharmacometric modeling [104,105]. Clinical experience further supports this concept. Successful treatment of severe CRKP infections has required therapeutic drug monitoring (TDM)-guided dose optimization in individual pediatric patients. At the same time, pharmacokinetic studies have demonstrated drug penetration characteristics that differ considerably from those predicted by adult models [106,107]. Collectively, these findings emphasize that insufficient pediatric PK/PD evidence remains a major barrier to optimizing antimicrobial therapy for biofilm-associated MDR infections [12].

#### 4.3.2. Anatomical Predispositions and Underlying Comorbidities

The prevalence of CAKUT—encompassing vesicoureteral reflux (VUR), uretero-pelvic junction obstruction, and other obstructive uropathies—is substantially higher in children than in adults. These conditions create a pathophysiologically favorable environment for biofilm establishment [108,109,110]. Urinary stasis, high-pressure bladder dynamics, and impaired mucosal defense mechanisms collectively promote bacterial adhesion and persistence, transforming the structurally abnormal urinary tract into a niche where *K. pneumoniae* biofilms can establish and recur with relative ease [38,108,111]. The epidemiological implication is direct: pediatric patients with urinary tract anomalies or neurogenic bladder are at heightened risk of recurrent and treatment-refractory UTIs, a population in which the limitations of both standard diagnostics and conventional therapy are most acutely felt [1,110,112]. In neonatal UTI cohorts, the identification of associated urinary tract anomalies has been highlighted as a key factor influencing both initial management and long-term follow-up strategies [12,113,114,115]. Multicenter surveillance from India has also documented that *K. pneumoniae* accounts for 15.7% of community-acquired uropathogens, with high-risk genomic clones—including ST15, ST16, and ST231—carrying multiple plasmids harboring co-resistance genes, indicating that resistant strains are no longer confined to the hospital environment [75]. Data from pediatric-specific surveillance studies in Saudi Arabia confirm *K. pneumoniae* as the second-most frequently isolated uropathogen in children, with MDR rates significantly higher in male patients and ESBL production increasingly prevalent [8].

#### 4.3.3. The Role of Medical Devices: Catheter-Associated UTIs

In pediatric and neonatal intensive care settings, urinary catheters are an indispensable clinical tool that simultaneously create one of the most favorable conditions for *K. pneumoniae* biofilm formation [14,116,117]. As already mentioned, the catheter surface provides an ideal abiotic substratum for type 3 fimbriae-mediated adhesion, and the resulting catheter-associated UTI (CAUTI) is architecturally distinct from mucosal infection and significantly more resistant to antibiotic clearance without device removal [118,119,120]. A prospective observational study conducted by González-Anleo et al. in a pediatric intensive care unit (PICU) found that 35.2% of CAUTI episodes were caused by MDR microorganisms, and that prior MDR colonization, prior MDR infection, and antibiotic exposure within the first 48 h of PICU admission were independently associated with MDR-CAUTI [118]. Critically, empirical treatment was inappropriate in 39.3% of MDR-CAUTI cases, compared with 7.1% in non-MDR cases, reflecting the inadequacy of current empirical strategies when confronted with resistant biofilm-forming pathogens in critically ill children [118]. Epidemiological data from Latin America further illustrate that pediatric patients exhibit a microbiological profile distinctly different from that of adults, with significantly higher proportions of *K. pneumoniae* and *Klebsiella* spp. among uropathogens, and that hospitalization, surgical service, and recent surgery are independent predictors of MDR/XDR infection [121].

Collectively, these findings underscore that device-associated *K. pneumoniae* biofilm infection in the pediatric intensive care setting represents a convergence of microbiological, pharmacological, and structural vulnerabilities that cannot be addressed solely by antibiotic selection. It also demands an integrated therapeutic and preventive framework, detailed in the following section.

## 5. Therapeutic Strategies and Future Directions

The mechanistic and clinical evidence reviewed in the preceding sections establishes a clear imperative: effective management of *K. pneumoniae* biofilm-associated UTI in children requires therapeutic strategies that target the biofilm community as an integrated biological structure, rather than its planktonic component alone. The following sections evaluate the principal approaches currently under investigation, examining the mechanistic rationale and available evidence for each, and identifying the translational, clinical, and regulatory barriers that impede their adoption in the pediatric population.

### 5.1. Catheter Stewardship and Dosing Optimization: Actionable Measures Pending Validated Diagnostics

No validated bedside test exists for biofilm-associated infection. In its absence, two interventions are already available to clinicians, supported by existing evidence. Both are independent of the diagnostic and therapeutic advances discussed in the remainder of this section. Unlike the strategies addressed below, they require no new technology. They can be implemented within current standards of care.

Prompt removal of urinary catheters is the first. When a catheter is no longer clinically required, its removal directly addresses the abiotic surface most favorable to the establishment of *K. pneumoniae* biofilms, as discussed in Section 4.3.3 [14,116,117]. Adherence to catheter-associated UTI prevention bundles reinforces this measure. These include aseptic insertion technique, closed drainage systems, and structured daily review of catheter necessity. Together, they remain among the few biofilm-prevention measures with direct pediatric applicability. None require specialized diagnostics [118].

Antimicrobial stewardship targeting sub-therapeutic dosing is the second lever. As described in Section 3.3, sub-inhibitory antibiotic concentrations induce adaptive upregulation of biofilm matrix genes, including pgaA and pgaB. This consolidates biofilm architecture rather than eradicating it. In pediatric patients, this risk is compounded by the PK/PD variability discussed in Section 4.3.1. Stewardship programs that avoid prolonged sub-therapeutic exposure, therefore, address a modifiable driver of both resistance and biofilm persistence. Such programs have already demonstrated reductions in MDR *K. pneumoniae* prevalence associated with stewardship [89,122,123,124,125].

Neither measure requires resolution of the diagnostic and translational gaps discussed throughout this review. They represent the most defensible point of clinical intervention available today. For that reason, they are presented first. The strategies that follow are mechanistically compelling. But they remain, to varying degrees, dependent on further preclinical and clinical development before pediatric practice can adopt them.

### 5.2. Optimized Antibiotic Combination Therapy

Combination antibiotic therapy currently represents the most readily applicable strategy for managing biofilm-associated *K. pneumoniae* infections. Unlike monotherapy, combination regimens are designed to overcome the multiple mechanisms responsible for biofilm-associated antimicrobial tolerance by simultaneously targeting distinct bacterial subpopulations and reducing the likelihood of resistance emergence [126,127,128].

The rationale for combination therapy is multifactorial. First, antibiotics with different physicochemical properties may achieve complementary penetration within the EPS matrix, thereby increasing overall antimicrobial exposure throughout the biofilm [65,129,130]. Second, combining agents with distinct mechanisms of action enables simultaneous activity against actively dividing bacteria and metabolically inactive persister cells, populations that coexist within mature biofilms and contribute to treatment failure [65,129,130]. Finally, exposure to multiple antibiotics targeting distinct cellular pathways reduces the likelihood of selecting for resistant subpopulations during therapy [131,132,133].

Among the available regimens, colistin-based combinations remain the most extensively investigated against CRKP biofilms [126,127,134,135,136]. Colistin increases outer membrane permeability, facilitating the penetration of companion antibiotics into the biofilm matrix. Synergistic activity has been reported with combinations of carbapenems, fosfomycin, rifampicin, and tigecycline. In contrast, the colistin–fosfomycin combination appears particularly promising because it combines membrane disruption with inhibition of cell-wall synthesis through complementary mechanisms [126,127,134,137,138]. Nevertheless, virtually all supporting evidence originates from in vitro biofilm models, and comparative clinical data remain unavailable.

Minocycline has recently emerged as another promising adjunctive agent, particularly in combination with carbapenems [119,139,140]. Experimental studies demonstrated reductions in biofilm biomass together with decreased expression of the efflux pump genes oqxA and oqxB, suggesting that its activity extends beyond conventional antibacterial effects and may interfere with biofilm-associated resistance mechanisms [119,140,141,142,143]. However, these findings have not yet been validated in pediatric clinical trials, and their clinical significance therefore remains uncertain.

The clinical application of combination therapy in children is further limited by age-dependent pharmacokinetic variability and drug-related toxicity. This is particularly relevant for colistin: Khamleh et al. found in a single-center cohort that nephrotoxicity reached 29.8% [99]. More studies report that robust pediatric dosing recommendations remain limited [100,101,102,103]. Consequently, current evidence supporting combination therapy in children relies predominantly on retrospective studies, case reports, and extrapolation from adult populations rather than randomized clinical trials.

New β-lactam/BLI combinations, particularly ceftazidime-avibactam (CZA), have considerably expanded treatment options for KPC- and OXA-48-producing CRKP; pediatric use is currently supported only by case-level evidence, including a TDM-guided regimen in a pediatric liver transplant recipient and a CZA-based regimen for pediatric CNS infection, rather than by prospective pediatric data [106,107]. Likewise, aztreonam-avibactam shows promising activity against metallo-β-lactamase-producing isolates in adult and in vitro studies, although pediatric clinical experience remains limited [12]. Despite these advances, current treatment guidelines continue to base antibiotic selection primarily on planktonic susceptibility testing. Few recommendations explicitly consider biofilm penetration or anti-biofilm activity when selecting combination regimens [144]. This disconnect between experimental evidence and clinical practice represents one of the principal obstacles to optimizing therapy for persistent pediatric *K. pneumoniae* infections.

Current evidence strongly supports combination therapy as the most rational pharmacological strategy for biofilm-associated CRKP infections. Nevertheless, almost all available data originate from experimental studies or adult populations, whereas prospective pediatric trials remain remarkably scarce. Consequently, the superiority of individual combinations cannot yet be established, emphasizing the need for pediatric PK/PD studies and randomized clinical trials specifically designed for biofilm-associated infections. Combination regimen-specific evidence and translational barriers are summarized alongside the other strategies reviewed in this section in Table 4.

### 5.3. Bacteriophage Therapy

Bacteriophage therapy has emerged as one of the most promising experimental strategies for treating biofilm-associated *K. pneumoniae* infections, owing to the highly specific interactions between bacteriophages and their bacterial hosts [146,147,148]. Unlike conventional antibiotics, bacteriophages replicate at the site of infection, and several phages targeting *K. pneumoniae* produce polysaccharide depolymerases that degrade both the bacterial capsule and the EPS matrix [149,150]. By disrupting the biofilm’s structural integrity, these enzymes facilitate phage penetration, increase bacterial exposure to host immune defenses, and enhance the activity of concurrently administered antimicrobial agents [149,150,151,152,153].

Current evidence suggests that the greatest therapeutic potential of phage therapy lies in combination approaches rather than phage monotherapy. Phage–antibiotic synergy (PAS), combinations with AMPs, and phage-loaded nanocarriers have consistently demonstrated greater biofilm disruption and bacterial killing than either intervention alone in experimental models [154,155,156]. These findings indicate that bacteriophages may function not only as direct antibacterial agents but also as biofilm-disrupting adjuvants that enhance the efficacy of conventional antimicrobial therapy.

An additional advantage of bacteriophage therapy is its high taxonomic specificity, which minimizes collateral damage to the commensal microbiota [157]. This characteristic may be particularly relevant in pediatric patients, in whom preservation of the developing intestinal microbiome is increasingly recognized as an important determinant of immune maturation and long-term metabolic health [157].

Despite these promising findings, important barriers currently limit clinical implementation. Most available data originate from in vitro studies or animal models, with isolated adult case-level evidence [145]. As stated in Section 5.2, combination antibiotic therapy has at least isolated pediatric case-level evidence. Bacteriophage therapy has not yet reached that threshold: no pediatric clinical studies exist, leaving this strategy at an earlier evidentiary stage than the pharmacological alternatives reviewed above. Furthermore, individualized phage selection, the potential emergence of phage-resistant bacterial variants, manufacturing standardization, and regulatory approval remain major challenges for routine clinical application [146,147,148,154,158,159]. Consequently, prospective evaluation is warranted before bacteriophage therapy can be considered an established therapeutic option for pediatric *K. pneumoniae* UTIs.

Future research should prioritize standardized phage susceptibility testing, evaluation of phage–antibiotic combinations in clinically relevant biofilm models, and well-designed pediatric clinical trials capable of defining the role of bacteriophage therapy in routine practice. Phage-based approaches are likewise summarized in Table 4.

### 5.4. Biofilm Matrix-Degrading Enzymes

Evidence for matrix-degrading enzymes targeting *K. pneumoniae* biofilms remains confined to in vitro systems, with no animal or human data reported to date. Biofilm matrix-degrading enzymes represent a mechanistically distinct antibiofilm strategy that targets EPSs rather than the bacterial cell itself. By disrupting the structural scaffold that supports biofilm stability, these agents enhance antibiotic penetration while simultaneously weakening bacterial defense mechanisms [160].

Among the most extensively investigated enzymes, DNase I and recombinant human DNase (dornase alfa) degrade extracellular DNA (eDNA), an essential structural component of mature biofilms that contributes to matrix stability, HGT, and antimicrobial tolerance [158,159]. Experimental studies have demonstrated that enzymatic degradation of eDNA improves antibiotic penetration and increases the susceptibility of biofilm-associated bacteria to agents such as colistin and aminoglycosides [161].

Another promising approach involves Dispersin B, a glycoside hydrolase that hydrolyzes poly-β-1,6-N-acetyl-D-glucosamine (PNAG), an important polysaccharide component of the *K. pneumoniae* biofilm matrix encoded by the pga operon [162]. Although Dispersin B exhibits only limited antibacterial activity as monotherapy, combination studies consistently demonstrate enhanced biofilm disruption and improved antimicrobial efficacy [162].

A major theoretical advantage of matrix-degrading enzymes is that they do not directly target bacterial viability and therefore are expected to exert considerably less selective pressure for resistance than conventional antibiotics. Nevertheless, almost all available evidence originates from in vitro investigations, whereas clinically relevant delivery strategies remain poorly defined. Intravesical administration appears biologically attractive but remains invasive, while systemic administration raises concerns regarding enzyme stability, tissue distribution, and potential off-target effects [163]. Consequently, despite their strong mechanistic rationale, the clinical applicability of matrix-degrading enzymes remains to be established, pending substantial translational development before pediatric clinical application [102,163].

### 5.5. Quorum Sensing Inhibition

Evidence for QS inhibition against *K. pneumoniae* biofilms is limited to in vitro and invertebrate model systems, with no human data reported to date. QS inhibition represents an anti-virulence strategy that aims to interfere with bacterial communication rather than directly eliminate bacterial cells [164]. By disrupting signaling pathways that regulate biofilm maturation, extracellular matrix production, and virulence factor expression, QS inhibitors (QSIs) may reduce biofilm formation while exerting less selective pressure for resistance than conventional bactericidal agents.

Given the central roles of the LuxS/AI-2 and SdiA pathways in biofilm regulation, as described previously, pharmacological disruption of QS signaling has emerged as an attractive upstream strategy capable of simultaneously modulating several determinants of biofilm-associated pathogenicity.

Several classes of QSIs—including furanone derivatives, plant-derived polyphenols such as quercetin, curcumin, and epigallocatechin gallate (EGCG), and synthetic AI-2 inhibitors—have demonstrated anti-biofilm activity in experimental models [165]. Among these, furanone C-30 combined with colistin has shown enhanced activity against colistin-resistant *K. pneumoniae* biofilms, reducing the antibiotic concentration required to eradicate biofilms [166,167].

Despite encouraging experimental findings, the clinical translation of QS inhibition remains limited. Considerable heterogeneity exists across published studies in terms of bacterial strains, experimental conditions, and outcome measures, making direct comparisons difficult. Moreover, pharmacokinetic data, urinary tract exposure, and pediatric safety profiles are currently unavailable, and no QS inhibitor has yet entered clinical evaluation for pediatric UTIs. Consequently, current evidence precludes routine implementation, and QS inhibition remains a promising experimental concept rather than a clinically applicable therapeutic strategy. From a pediatric urinary tract standpoint, this limitation is compounded further: QS inhibition depends on sustained sub-MIC mucosal exposure, which is difficult to reconcile with the intermittent voiding pattern characteristic of infants and toddlers, and the cytotoxicity documented for furanone-class compounds raises additional concern for any bladder-instillation application in this age group.

### 5.6. Antimicrobial Peptides

Evidence for antimicrobial peptides against *K. pneumoniae* biofilm remains confined to in vitro systems, with no animal model or human data reported to date. AMPs constitute a diverse group of naturally occurring and synthetic molecules that exert rapid bactericidal activity primarily by disrupting bacterial membranes. This mechanism largely bypasses conventional resistance pathways [168,169]. In addition to their direct antibacterial effects, several AMPs can penetrate biofilms and retain activity against metabolically inactive persister cells. These two characteristics distinguish them from many conventional antibiotics [170,171].

Experimental studies have identified several promising candidates, including polymyxin-derived peptides, LL-37, and synthetic AMP analogs engineered to improve antibacterial activity while reducing toxicity [170,171]. Because AMPs combine membrane disruption with biofilm penetration, they may complement rather than replace existing antimicrobial therapy.

Intravesical AMP delivery has been proposed as an attractive strategy for pediatric UTIs because it could achieve high local drug concentrations while minimizing systemic exposure and toxicity [172,173]. However, virtually all currently available evidence remains preclinical, and important questions regarding peptide stability, urinary persistence, manufacturing costs, and large-scale clinical applicability remain unresolved. No AMP has yet progressed to clinical evaluation for pediatric biofilm-associated UTIs. In addition, intravesical retention time, which is a key determinant of AMP efficacy, is difficult to control in children who are not yet toilet-trained, and the susceptibility of peptides to proteolytic degradation in urine further constrains their practical translational prospects in this population, specifically, independent of the broader preclinical status of the field.

Table 4 summarizes the anti-biofilm mechanisms, current evidence levels, and principal translational barriers of the therapeutic strategies reviewed above.

Taken together, these emerging strategies target distinct components of biofilm biology rather than solely bacterial viability. However, despite encouraging preclinical results, none have yet generated sufficient pediatric clinical evidence to support routine implementation. Consequently, optimized antibiotic therapy remains the current standard of care, whereas non-antibiotic approaches should presently be considered adjunctive strategies requiring further translational validation.

### 5.7. Nanoparticle-Based Anti-Biofilm Delivery Systems

Evidence for nanoparticle-based anti-biofilm strategies against *K. pneumoniae* remains confined to in vitro systems, with no animal-model or human data reported to date. Nanotechnology-based platforms constitute a further emerging strategy against *K. pneumoniae* biofilms, acting through mechanisms distinct from those of conventional antibiotics, phage-derived enzymes, or QS inhibition. Metallic nanoparticles, particularly biogenic silver nanoparticles synthesized via green chemistry methods, have demonstrated both direct antibacterial activity and anti-biofilm efficacy against carbapenem-resistant *K. pneumoniae*, with proposed mechanisms including reactive oxygen species generation, membrane disruption, and downregulation of biofilm-associated genes such as mrkA and luxS [96]. Zinc-based nanoparticle formulations have shown comparable anti-biofilm activity in experimental models, inhibiting biofilm formation and reducing established biofilm biomass in a dose-dependent manner [174].

Lipid-based nanocarriers offer a complementary approach by improving the delivery of conventional antibiotics rather than acting as independent antimicrobials. Niosomal encapsulation of gentamicin combined with EDTA has been shown to enhance antibacterial and anti-biofilm activity against catheter-associated *K. pneumoniae* isolates, with concurrent downregulation of biofilm-related genes, illustrating the particular relevance of this delivery strategy for device-associated pediatric infections [175]. Similarly, nanocomposite coatings—most extensively studied using titanium dioxide—have been proposed as a preventive strategy for catheter and medical device surfaces, reducing biofilm establishment by carbapenemase-producing *K. pneumoniae* before infection occurs [176].

Despite these encouraging preclinical findings, nanoparticle-based strategies remain at an early stage of translational development. Concerns regarding cytotoxicity, environmental persistence, and the biological behavior of nanomaterials in immature organ systems have not been systematically addressed in pediatric populations, and no nanoparticle-based anti-biofilm formulation has entered clinical evaluation for pediatric *K. pneumoniae* UTI. Given the substantial preclinical literature specifically targeting *K. pneumoniae* biofilm, dedicated pediatric toxicological and pharmacokinetic studies represent a necessary prerequisite before clinical translation can be considered. This is a more fundamental barrier for nanomaterials than for pharmacologically conventional agents: renal and urothelial tissues remain structurally immature in young children, and the long-term tissue accumulation and clearance kinetics of nanoparticles in a developing urinary system are entirely unstudied, raising a distinct safety question rather than merely an evidentiary gap.

### 5.8. The Urobiome and Prophylactic Strategies

Unlike the preceding non-antibiotic strategies, urobiome modulation has reached limited human trial data, though none specific to *K. pneumoniae*. The urobiome has recently emerged as a potential determinant of susceptibility to recurrent UTIs and biofilm establishment. Increasing evidence suggests that disruption of the normal urinary microbial community, particularly the depletion of Lactobacillus spp. and other protective commensals, may facilitate colonization by opportunistic uropathogens, including *K. pneumoniae* [177,178]. However, the specific interactions between the pediatric urobiome and *K. pneumoniae* biofilm formation remain poorly understood, and causality has not yet been established [157].

Consequently, modulation of the urobiome through probiotics or other microbiome-directed interventions has attracted increasing interest as a non-antibiotic preventive strategy. Unlike antimicrobial prophylaxis, these approaches may reduce recurrent infections without increasing selective pressure for multidrug resistance. Nevertheless, current pediatric evidence remains limited, heterogeneous, and insufficient to support routine clinical implementation. Future studies should clarify which patient populations are most likely to benefit and identify microbial signatures associated with protection against recurrent biofilm-associated UTIs. This uncertainty is compounded by the substantial age-dependent variability of the urobiome itself, including the hormonally driven shift toward Lactobacillus predominance around puberty; probiotic strategies validated in adult populations cannot therefore be assumed to transfer directly to prepubertal children.

### 5.9. The Guideline Gap and the Imperative for Translational Research

Despite the growing understanding of biofilm biology and the increasing number of experimental anti-biofilm strategies, a substantial gap persists between mechanistic knowledge and routine pediatric clinical practice. Current international recommendations, including the updated 2025 EAU/ESPU guidelines, continue to base therapeutic decisions primarily on conventional urine culture and planktonic antimicrobial susceptibility testing [144]. Neither biofilm-specific diagnostic approaches nor biofilm-guided therapeutic algorithms are currently incorporated into pediatric UTI management, and multidrug-resistant biofilm-forming *K. pneumoniae* is not considered a distinct clinical entity requiring specific recommendations.

This omission is not an oversight but a direct consequence of the evidence hierarchy guideline bodies require. Recommendation-issuing panels, such as those that inform AAP and NICE pediatric UTI guidance, typically require validated diagnostic criteria and randomized or comparative-effectiveness data before endorsing a management pathway [1,19]. As established in Section 4.1, no biofilm-specific diagnostic test has been validated for bedside use, and, as Table 4 illustrates, virtually every antibiofilm strategy remains at the case series or preclinical stage. In the absence of a way to identify the target population and a controlled-trial evidence base demonstrating benefit, guideline bodies have no defensible basis on which to issue biofilm-specific recommendations, regardless of the mechanistic plausibility reviewed here.

Bridging this gap requires coordinated translational research spanning experimental, pharmacological, and clinical investigation. Four priorities are particularly urgent. Validated biomarkers or diagnostic assays are needed to identify biofilm-associated infection in individual pediatric patients, since no current bedside test exists. Biofilm diagnostic methodology must be standardized across research groups; without this, trial populations cannot be consistently defined. Dedicated pediatric PK/PD studies are needed for agents with demonstrated anti-biofilm activity, given that data are absent or partial for nearly every strategy reviewed. Also, randomized pediatric clinical trials must be built on these diagnostic criteria once established, rather than extrapolated from adult or in vitro models. Preclinical models that better reflect the pediatric urinary tract are needed to evaluate emerging antibiofilm therapies under clinically relevant conditions. The pediatric PK/PD gap for newer agents such as CZA and aztreonam-avibactam, discussed previously, exemplifies the broader translational deficit this review has identified and should be treated as a priority within any coordinated research agenda [12]. Most importantly, prospective pediatric clinical trials evaluating both optimized combination therapy and novel anti-biofilm interventions remain largely absent, limiting the development of evidence-based recommendations.

Section 5.1 established that catheter stewardship and dosing optimization already offer actionable, evidence-supported interventions. These do not depend on the translational advances discussed above. The principal remaining gap is not evidentiary. It is the formal integration of these measures into pediatric guidelines.

Taken together, these strategies target distinct components of biofilm biology rather than solely bacterial viability, yet each faces a translational barrier specific to the pediatric urinary tract rather than a uniform lack of pediatric data: QS inhibition is constrained by voiding-dependent mucosal exposure, AMPs by urinary proteolysis and bladder retention time, and the nanoparticle- and urobiome-directed strategies discussed below by tissue immaturity and age-dependent microbial variability, respectively. Consequently, optimized antibiotic therapy remains the current standard of care. In contrast, non-antibiotic approaches should presently be considered adjunctive strategies requiring translational validation tailored to these pediatric-specific constraints, rather than simple extrapolation from adult or in vitro data.

## 6. Limitations

Several limitations of this review should be acknowledged. First, as a narrative rather than a systematic review, study selection was investigator-driven rather than governed by a predefined, reproducible protocol; while appropriate for synthesizing a mechanistically and clinically heterogeneous literature, this approach carries an inherent risk of selection bias that a systematic or scoping methodology would more directly mitigate. Second, the search was restricted to English-language publications, which may have excluded relevant regional or non-English clinical data, particularly given that pediatric *K. pneumoniae* UTI epidemiology varies substantially by geographic region and healthcare setting. Third, given the scarcity of pediatric-specific mechanistic data, this review necessarily incorporated substantial evidence from adult and in vitro studies. However, this evidence is identified as such throughout; its extrapolation to pediatric populations should be interpreted with appropriate caution.

## 7. Conclusions

Biofilm-associated *K. pneumoniae* UTIs in children represent a complex clinical and microbiological challenge, reflecting the interplay between bacterial adaptation and the physiological particularities of the pediatric host. Biofilms function not merely as physical barriers but as dynamic ecosystems that integrate impaired antibiotic penetration, metabolic persistence, HGT, and adaptive stress responses, thereby promoting antimicrobial tolerance and facilitating the selection and dissemination of multidrug-resistant strains.

The principal contribution of this review lies not in the mechanistic evidence itself, which is largely established in the broader *K. pneumoniae* biofilm literature, but in its integration into a pediatric clinical and translational framework that remains comparatively underdeveloped. By systematically connecting biofilm biology to pediatric-specific diagnostic limitations, pharmacokinetic constraints, and device- and anomaly-related risk factors, this review identifies where existing knowledge can already inform pediatric practice and where it cannot yet be safely extrapolated.

This integrative perspective clarifies that successful management of biofilm-associated *K. pneumoniae* UTIs in children will require biofilm-informed therapeutic strategies specifically adapted to the pediatric population, rather than direct extrapolation of adult-derived evidence. The evidence base underlying most of these strategies remains at the level of case series, retrospective cohorts, or preclinical models, with pediatric PK/PD data largely absent; this evidence gap, rather than a lack of mechanistic rationale, is the principal barrier to clinical translation. In the interim, catheter stewardship and dosing optimization remain the most defensible points of clinical intervention.

Future progress will depend on the development of clinically relevant pediatric biofilm models, dedicated PK and PD studies, standardized diagnostic methods capable of identifying biofilm-associated infections, and well-designed prospective clinical trials evaluating emerging anti-biofilm therapies. We propose that future pediatric guidelines should progressively incorporate biofilm biology into therapeutic decision-making as this evidence matures, enabling treatment strategies to evolve beyond conventional susceptibility testing toward a more individualized, mechanistically informed approach.

## Figures and Tables

**Table 1:** Mechanisms of biofilm-mediated antibiotic resistance in *Klebsiella pneumoniae:* clinical implications and corresponding therapeutic targets.

Mechanism	Clinical Implication	Therapeutic Target
EPS diffusion barrier	Antibiotic concentrations at biofilm depth fall below MIC despite adequate serum levels; in vitro susceptibility does not predict clinical outcome	Matrix disruption (DNase I, dispersin B, phage depolymerases); agents with superior EPS penetration (fosfomycin, rifampicin)
Persister cell induction	Infection relapses after cessation of therapy despite apparent microbiological cure; may contribute to unreliable prediction of treatment response	Agents active under metabolic quiescence (AMPs, certain QSIs); combination regimens targeting heterogeneous physiological states
SOS-mediated adaptive mutagenesis	Sub-inhibitory antibiotic exposure accelerates the emergence of heritable resistance mutations within the biofilm	Avoidance of prolonged sub-therapeutic dosing; combination therapy to suppress mutant selection window
HGT amplification	Biofilm functions as a resistance gene dissemination reservoir; ESBL and carbapenemase determinants spread between co-resident organisms	Biofilm disruption as an infection control imperative, not merely a therapeutic goal; stewardship to limit selective pressure driving co-resistance acquisition
QS coordination	Biofilm maturation, EPS production, and virulence factor expression are density-dependent and collectively regulated; disruption of one QS axis attenuates multiple downstream resistance determinants	QS inhibition (LuxS/AI-2 and SdiA axes) as an upstream intervention capable of simultaneously attenuating multiple biofilm-mediated pathogenicity determinants

Rifampicin is included in the table based on its favorable EPS matrix penetration rather than as a first-line agent for *K. pneumoniae* UTIs; its rapid selection for resistance when used as monotherapy limits its clinical role to combination regimens targeting biofilm-embedded organisms.

**Table 2:** Diagnostic approaches for biofilm-associated *K. pneumoniae* UTIs in children: limitations, clinical implications, and current clinical status.

Diagnostic Approach	Main Limitation	Clinical Implication	Current Status
Urine culture	Detects only planktonic bacteria	False-negative cultures	Routine practice
MIC testing	Does not reflect biofilm susceptibility	Underestimates required antibiotic concentrations	Routine practice
16S rRNA sequencing	Limited specificity	Adjunctive diagnosis	Limited clinical use
CLSM	Research only	Direct biofilm visualization	Experimental
Congo red agar	Semi-quantitative	Biofilm screening	Experimental
Microtiter biofilm assay	Quantifies biofilm biomass	Research tool	Experimental

**Table 3:** Clinical challenges to effective antibiotic therapy in pediatric *K. pneumoniae* UTIs and their implications for management.

Challenge	Evidence	Clinical Implication
Biofilm-mediated tolerance	EPS + persisters	Reduced antibiotic efficacy
Increasing MDR prevalence	Global surveillance	Limited empirical therapy
ESBL production	Rising incidence	Carbapenem dependence
TMQR	Coexists with ESBL	Limited fluoroquinolone utility
CRKP	NICU dissemination	Few therapeutic options
Pediatric PK limitations	Age-dependent	Restricted antibiotic choices

TMQR, transferable mechanisms of quinolone resistance.

**Table 4:** Therapeutic strategies targeting *Klebsiella pneumoniae* biofilm in pediatric UTIs: anti-biofilm mechanisms, evidence levels, and translational barriers.

Agent/Class	Anti-Biofilm Mechanism	Best Evidence Level	Pediatric PK/PD Data	Primary Translational Barrier
Colistin-based combinations	Outer membrane permeabilization; enhances co-agent EPS penetration; dual-target synergy	Case series/retrospective cohort	Partial	Nephrotoxicity up to 29.8% in one pediatric cohort *; no validated dosing algorithm; no pediatric RCTs
Minocycline + carbapenem	Suppresses oqxA/oqxB efflux pumps; increases membrane permeability; reduces biofilm biomass	In vitro	Partial	Preclinical only; anti-biofilm dosing undefined; no trial data
CZA (β-lactam/BLI)	Restores KPC/OXA-48 coverage via β-lactamase inhibition; biofilm penetration uncharacterized	Case series/compassionate use	Partial	Limited pediatric PK; adult dosing not extrapolable; no biofilm-informed guidance
Aztreonam-avibactam (β-lactam/BLI)	Extends coverage to MBL producers; potential depolymerase synergy	Case series/compassionate use	None (neonates/infants)	Critically scarce neonatal/infant data; no pediatric RCT; regulatory pathway undefined
Lytic phages (monotherapy)	Direct bacterial lysis; narrow host-range killing of actively replicating cells	Case report (adult; clinical registered trial) **	None	Requires individualized strain-matching; resistance can emerge within hours
Phage depolymerases	Degrades capsule/EPS matrix; restores susceptibility independent of phage viability	In vitro/animal model	None	Requires rapid strain-matching; no pediatric RCT; no compassionate-use pathway in most jurisdictions
DNase I/dornase alfa	Degrades eDNA matrix component; sensitizes organisms to colistin/aminoglycosides	In vitro	None	Requires intravesical delivery; no pediatric protocol; systemic biodistribution uncharacterized
Dispersin B	Degrades PNAG (pga operon); reduces biomass in combination only; no monotherapy bactericidal activity	In vitro	None	No clinical-stage formulation; delivery route and regulatory pathway undefined
QSIs	Disrupts LuxS/AI-2 and SdiA signaling; attenuates EPS/fimbriae expression without bactericidal pressure	In vitro/invertebrate model	None	No human urinary-tract PK data; no UTI trials (adult or pediatric); reduced resistance pressure unproven in vivo
AMPs	Membrane disruption independent of resistance pathways; penetrates EPS matrix; active against quiescent persisters	In vitro	None	No clinical-stage formulation for pediatric UTI; proposed intravesical delivery unvalidated; pediatric toxicity uncharacterized
Probiotics/urobiome modulation	Restores Lactobacillus-dominant urobiome; competes against uropathogen colonization	RCT (limited; not *K. pneumoniae*-specific)	Partial	Link to *K. pneumoniae* biofilm susceptibility incompletely characterized; insufficient evidence for recommendations

Abbreviations: AMP, antimicrobial peptide; BLI, β-lactamase inhibitor; eDNA, extracellular DNA; EGCG, epigallocatechin gallate; EPS, extracellular polymeric substance; KPC, *K. pneumoniae* carbapenemase; MBL, metallo-β-lactamase; NDM, New Delhi metallo-β-lactamase; OXA-48, oxacillinase-48; PAS, phage–antibiotic synergy; PK/PD, pharmacokinetic/pharmacodynamic; PNAG, poly-N-acetylglucosamine; QSI, quorum-sensing inhibitor; RCT, randomized controlled trial; UTI, urinary tract infection. * Single-center pediatric cohort [99]. ** Registered clinical trial for an adult case report [145].

## Data Availability

No new data were created or analyzed in this study. Data sharing is not applicable to this article.

## Abbreviations

The following abbreviations are used in this manuscript:

UTIs	Urinary Tract Infections
MDR	Multidrug-Resistant
HGT	Horizontal Gene Transfer
*K. pneumoniae*	*Klebsiella pneumoniae*
QSIs	QS inhibitors
AMPs	Antimicrobial Peptides
*E. coli*	*Escherichia coli*
ESBLs	Extended-Spectrum β-Lactamases
EPS	Extracellular Polymeric Substance
CAKUT	Congenital Anomalies Of The Kidney And Urinary Tract
QS	Quorum Sensing
MIC	Minimum Inhibitory Concentration
DNA	Deoxyribonucleic Acid
KPC	*Klebsiella pneumoniae* Carbapenemase
NDM	New Delhi Metallo-β-Lactamase
OXA-48	Oxacillinase-48
XDR	Extensively Drug-Resistant
AI-2	Autoinducer-2
PNAG	Poly-N-Acetylglucosamine
hvKP	Hypervirulent *K. pneumoniae*
cKP	Classical *K. pneumoniae*
CRKP	Carbapenem-Resistant *K. pneumoniae*
RNA	Ribonucleic Acid
CLSM	Confocal Laser Scanning Microscopy
NICU	Neonatal Intensive Care Unit
TMQR	Transferable Mechanisms Of Quinolone Resistance
PK/PD	Pharmacokinetic/Pharmacodynamic
BLI	β-Lactamase Inhibitor
TDM	Therapeutic Drug Monitoring
VUR	Vesicoureteral Reflux
CAUTI	Catheter-Associated UTI
PICU	Pediatric Intensive Care Unit
CZA	Ceftazidime-Avibactam
PAS	Phage–Antibiotic Synergy
eDNA	Extracellular DNA
EGCG	Epigallocatechin Gallate
MBL	Metallo-β-Lactamase
EAU	European Association of Urology
ESPU	European Society for Paediatric Urology
